# The Membrane-Anchoring Region of the AcMNPV P74 Protein Is Expendable or Interchangeable with Homologs from Other Species

**DOI:** 10.3390/v13122416

**Published:** 2021-12-02

**Authors:** María Victoria Nugnes, Alexandra Marisa Targovnik, Adrià Mengual-Martí, María Victoria Miranda, Carolina Susana Cerrudo, Salvador Herrero, Mariano Nicolás Belaich

**Affiliations:** 1 Laboratorio de Ingeniería Genética y Biología Celular y Molecular—Área Virosis de Insectos, Instituto de Microbiología Básica y Aplicada, Departamento de Ciencia y Tecnología, Universidad Nacional de Quilmes, Roque Sáenz Peña 352, Bernal, Buenos Aires B1876BXD, Argentina; victorianugnes@gmail.com (M.V.N.); ccerrudo@unq.edu.ar (C.S.C.); 2 Cátedra de Biotecnología, Departamento de Microbiología, Inmunología, Biotecnología y Genética, Facultad de Farmacia y Bioquímica, Universidad de Buenos Aires, Junín 956, Sexto Piso, Buenos Aires C1113AAD, Argentina; alexandra.targovnik@gmail.com (A.M.T.); mvic@ffyb.uba.ar (M.V.M.); 3 Instituto de Nanobiotecnología (NANOBIOTEC), Universidad de Buenos Aires (UBA)—Consejo Nacional de Investigaciones Científicas y Técnicas (CONICET), Junín 956, Sexto Piso, Buenos Aires C1113AAD, Argentina; 4 Department of Genetics and University Institute of Biotechnology and Biomedicine-BIOTECMED, Universitat de València, Dr. Moliner 50, Valencia, 46100 Burjassot, Spain; adria.mengual@uv.es (A.M.-M.); Salvador.Herrero@uv.es (S.H.)

**Keywords:** baculovirus, P74, AcMNPV, SeMNPV, HearSNPV, CRISPR/Cas9, *Spodoptera exigua*, *Rachiplusia nu*

## Abstract

Baculoviruses are insect pathogens that are characterized by assembling the viral dsDNA into two different enveloped virions during an infective cycle: occluded virions (ODVs; immersed in a protein matrix known as occlusion body) and budded virions (BVs). ODVs are responsible for the primary infection in midgut cells of susceptible larvae thanks to the per os infectivity factor (PIF) complex, composed of at least nine essential viral proteins. Among them, P74 is a crucial factor whose activity has been identified as virus-specific. In this work, the *p74* gene from AcMNPV was pseudogenized using CRISPR/Cas9 technology and then complemented with wild-type alleles from SeMNPV and HearSNPV species, as well as chimeras combining the P74 amino and carboxyl domains. The results on *Spodoptera exigua* and *Rachiplusia nu* larvae showed that an amino terminal sector of P74 (lacking two potential transmembrane regions but possessing a putative nuclear export signal) is sufficient to restore the virus infectivity whether alone or fused to the P74 transmembrane regions of the other evaluated viral species. These results provide novel information about the functional role of P74 and delimit the region on which mutagenesis could be applied to enhance viral activity and, thus, produce better biopesticides.

## 1. Introduction

The *Baculoviridae* family is composed of insect-specific rod-shaped viruses with large covalently closed circular double-stranded DNA (cccdsDNA), from 81,755 bp (NeleNPV) to 178,733 bp (XecnGV) in length, usually used as crop pest control agents among other biotechnological uses [1,2]. In nature, baculoviruses infect susceptible hosts in the larval state and produce two genetically identical but morphologically distinct viral phenotypes: occlusion-derived viruses (ODVs) and budded viruses (BVs) [3]. The first are those that initiate the infection in the midgut defining the host range (primary infection), and they are characterized by being embedded in a crystalline matrix denominated occlusion bodies (OBs) that physically protect virions against environmental stresses, while the BVs are responsible for cell-to-cell infections inside the larval body (secondary or systemic infection) [4].

Baculoviruses are classified into four genera according to their nucleotide sequence and phylogeny [5], permissive host species [6], and the type of main OB protein [7]: *Alphabaculovirus*, *Betabaculovirus*, *Gammabaculovirus*, and *Deltabaculovirus*. While alpha- and betabaculoviruses infect insects belonging to Lepidoptera order, gamma- and deltabaculoviruses infect members of the Hymenoptera and Dyptera orders, respectively [3]. Furthermore, the *Alphabaculovirus* genus is subclassified into two clades, “group I” and “group II”, according to phylogeny inferences and the occurrence of different envelope fusion proteins in BVs [8]. The GP64 protein is present in group I alphabaculoviruses [9], whereas, for all the others, including those belonging to group II, the F protein is the factor associated with cell-to-cell spreading [10,11,12].

Baculovirus infection starts with the ingestion of OB-contaminated food (per os route) by the larvae [13]. Then, OBs get dissolved in the alkaline larval midgut and release the ODVs into the gut lumen to infect the epithelial cells. Once ODVs pass through the peritrophic matrix, the viral envelope fuses with the cell membrane, releasing the nucleocapsids. This process of recognition, binding, and fusion determines the success of the infection, and it is carried out by a group of viral proteins anchored in the ODV envelope, termed per os infectivity factors (PIFs) [14,15,16,17]. So far, 10 PIFs have been identified: PIF0 (Ac138), originally called P74 because of its molecular weight of 74 kDa [18,19], PIF1 (Ac119) [20], PIF2 (Ac22) [21,22], PIF3 (Ac115) [23,24], PIF4 (Ac96) [25,26], PIF5 (ODV-E56 or Ac148) [27,28], PIF6 (Ac68) [29], PIF7 (Ac110) [30], PIF8 (VP91 or Ac83) [31,32], and PIF9 (Ac108) [33,34]. Interestingly, except for PIF9 which has no orthologs detected in *Deltabaculovirus* [35], all the genes that encode these factors are part of the 38 core genes described until now in *Baculoviridae* [31] and are also conserved in other invertebrate DNA viruses, such as nudiviruses, hytrosaviruses, bracoviruses, and nimaviruses [3,36]. In accordance with the information reported, all the PIF proteins except for PIF5 are arranged in a complex, in which PIF1, 2, 3, and 4 form a stable core and PIF0 (P74), 6, 8, and 9 are more loosely associated [17,34,37]. When any of these factors is deleted from the baculoviral genome, it results in the loss of oral infectivity by the inhibition of the primary infection [36,38]. However, the infective capacity of BVs does not seem diminish, since PIFs are not needed for cell-to-cell spreading. Consequently, in vitro propagation in insect cell culture of a PIF-deficient baculovirus is not affected and shows wild-type characteristics, thus facilitating the study of these variants [20,21,22].

P74 was the first viral protein found to be essential for baculovirus oral infection and, more recently, has also been described to play a fundamental role in the ODV binding to larvae’s midgut epithelium [16,39]. The N-terminus portion of the protein is directed outward from the ODV envelope, and, unlike PIF1–4, 7, and 8, an inner nuclear membrane sorting motif (INM-SM) has not been described [38,40,41]. On the other hand, the C-terminus is believed to be anchored in the ODV envelope, and whether it contains of two or three transmembrane (TM) domains is still under discussion [38,42]. It has been reported that P74 of *Autographa californica* nucleopolyhedrovirus (AcMNPV) undergoes two independent cleavages once the OB dissolves in the midgut lumen. The first is mediated by a host-derived protease and divides the protein into two fragments of approximately 35 and 40 kDa. The second cleavage is executed by insect midgut trypsins, releasing a 20 kDa fragment from the N-terminus, which still interacts with the rest of the PIF complex [43]. This process would be the crucial step to activate P74 [44]. Moreover, other studies have demonstrated that the infection of susceptible larvae with a *p74*-knockout AcMNPV could be rescued by cofeeding with the purified protein or even with a soluble truncated form without the C-terminal TM domain (1–580 aa) [42,45]. Nonetheless, previous attempts to replace P74 with other homologs have failed, resulting in the completely loss of the oral infectivity. This highlights the highly specific interactions among PIF proteins and between these and the cellular factors of the midgut epithelial cells to support the primary infection [46,47].

Manipulation through viral genome engineering such as PIF deletions or modifications allows us to assess the protein function and its implications in the baculoviral infectivity. In recent years, genome mutagenesis has become easier with the introduction of clustered regularly interspaced short palindromic repeats/Cas9 or CRISPR/Cas9 technology [48,49,50]. The CRISPR/Cas9 system involves a single guide RNA (sgRNA) that interacts and directs the Cas9 endonuclease to a specific site to perform a double-strand DNA break (DSB) [51]. This triggers DNA repair via one of two major pathways, the error-prone nonhomologous end joining (NHEJ) or the homology-directed repair (HDR) pathway. The latter mechanism requires the provision of donor DNA with homology to the flanking sequence of the DSB. However, in the absence of this template, the endogenous cell repair machinery conducts NHEJ, leaving consequential insertions/deletions (indel) mutations as a scar near the site of the break [52].

In this work, a variant of AcMNPV, the prototypic baculovirus most used for biotechnological purposes, was knocked out in the *p74* gene by CRISPR/Cas9 technology and NHEJ repair, and then complemented with sequences that encode P74 domains or P74 chimeras generated by combinations of domains from different baculovirus species (*Spodoptera exigua* multiple nucleopolyhedrovirus (SeMNPV) and *Helicoverpa armigera* single nucleopolyhedrovirus (HearSNPV)). The characterization of the P74 protein and the biological effects on susceptible larva add new evidence for understanding the baculovirus PIF complex.

## 2. Materials and Methods

### 2.1. In Vitro Cell Culture and Insect Rearing

The *Spodoptera frugiperda* Sf21 insect cell line [53] (obtained from GIBCO BRL, Gaithersburg, MD, USA) was cultured on monolayers at 25 °C in Grace’s medium (GIBCO BRL, Gaithersburg, MD, USA) supplemented with 10% fetal bovine serum (FBS; GIBCO BRL, Gaithersburg, MD, USA) and antibiotics and antimycotics (GIBCO BRL Gaithersburg, MD, USA). *Spodoptera exigua* larvae were derived from the established colony at the University of Valencia (Spain), and *Rachiplusia nu* larvae were obtained from AgIdea S.A. (Pergamino, Buenos Aires, Argentina). Insects were reared with an artificial diet at 25–27 °C with 70% relative humidity and a photoperiod of 16/8 h (light/dark).

### 2.2. Bioinformatics Studies

A nonredundant set of 76 P74 protein sequences was obtained from the reported genomes (22 group I alphabaculoviruses, 28 group II alphabaculoviruses, 22 betabaculoviruses, three gammabaculoviruses, and 1 deltabaculovirus) (Supplementary Table S1) and aligned using ClustalX [54]. The consensus line was used to generate a sequence similarity plot employing a residue-by-residue sliding window strategy (35 nucleotides long), where arbitrary values were assigned to different symbols (+1 for identical residues, +0.5 and +0.25 for different degrees of conservative changes, and −0.2 for nonconservative changes). The algebraic sum was normalized by the window length and allotted to the window central position. Moreover, the evolutionary history was inferred with MEGA7 [55] using the maximum likelihood method based on the LG + G + I model [56], while also considering orthologs to P74 of nudiviruses, hytrosaviruses, and nimaviruses (Supplementary Table S1). Initial trees for the heuristic search were obtained using neighbor-joining and BioNJ algorithms (the matrix of pairwise distances was estimated using a JTT model), and the topology with a superior log likelihood value was selected. A discrete gamma distribution was used to model the different evolutionary rates among sites (two categories (+G, parameter = 1.1482)). The weblogo server was used with default parameters (https://weblogo.berkeley.edu/logo.cgi; last access 1 October 2021) [57], and the hydropathy profiles were generated using a sliding overlapping window strategy (21 residues long displaced from one residue at a time). The putative transmembrane domains, the disulfide bonds, and the nuclear export signal (NES) predictions were performed using webservers (https://services.healthtech.dtu.dk/service.php?TMHMM-2.0, accessed on 26 November 2021, [58]; http://disulfind.disi.unitn.it/, accessed on 26 November 2021, [59]; http://clavius.bc.edu/~clotelab/DiANNA/, accessed on 26 November 2021, [60]; https://services.healthtech.dtu.dk/service.php?NetNES-1.1 [61]; last access 1 October 2021) and standard parameters.

### 2.3. P74 Gene Knockout in AcMNPV-bacmid

The AcMNPV-bacmid bMON14272 [62] was depleted in the *p74* gene using CRISPR/Cas9 technology. Briefly, the CRISPR RNA (crRNA, 5′ AACTGGCTTTCAGCAAGCGC 3′) used to direct the *Streptococcus pyogenes* Cas9 nuclease (New England Biolabs, Ipswich, MA, USA) to the initial part of the *p74* open reading frame (ORF) was previously designed 207 bp downstream of the ATG codon [50] using the CHOP-CHOP online platform (https://chopchop.rc.fas.harvard.edu accessed on 26 November 2021) and synthesized (Integrated DNA Technologies, Leuven, Belgium). To generate the ribonucleoprotein (RNP) complex, 1 μL of the crRNA (1 μM) was incubated with 1 μL of tracrRNA (1 μM) and 98 μL of nuclease-free duplex buffer (both from Integrated DNA Technologies, Leuven, Belgium) for 5 min at 95 °C. Then, 1 μL of the crRNA–tracrRNA mixture was combined with 6.2 μL of Cas9 (1 mM) and 2.8 μL of nuclease-free water to a final volume of 10 μL (incubation at 37 °C for 5 min). The bacmid DNA was purified following the manufacturer’s instructions of Bac-to-Bac system (GIBCO BRL Gaithersburg, MD, USA) and added to the previous mixture (1 μg), which was then transfected in Sf21 cells (70% confluent) using Cellfectin^®^ II Reagent (Invitrogen, Carlsbad, CA Carlsbad, USA) in Grace’s medium without FBS. After 5 h of incubation, the medium was replaced by Grace’s medium supplemented with 10% FBS and incubated for 72 h at 25 °C. Later, BVs were isolated from supernatants following the plaque assay protocol [63], and the individual clones were analyzed by amplicon Sanger sequencing using PrepMan^®^ Ultra reagent (Applied Biosystems, Foster City, CA, USA) and the primers FwP74seq and RvP74seq (Table 1) [50] to detect indel mutations. Once P74 depleted bacmid (bMON14272Δp74) was identified, BVs were replicated in Sf21 cells, and the viral DNA was purified using the standard phenol/chloroform protocol [63] to be sequenced by Illumina (MiSeq System, Laboratorio de Secuenciación Servicio Central de Soporte a la Investigación-SCSIE, Universitat de València, Valencia, Spain). Finally, DH10b *Escherichia coli* competent cells were transformed with the AcMNPV-bacmidΔp74 DNA and the pMON7124 helper plasmid (GIBCO BRL Gaithersburg, MD, USA) to generate the DH10bacΔp74 strain for further Bac-to-Bac applications.

### 2.4. AcMNPV-bacmidΔp74 Complementation

According to the bioinformatics analysis of the P74 protein, several baculovirus-transfer plasmids were constructed in pFastBac-Dual vector (GIBCO BRL Gaithersburg, MD, USA) carrying the green fluorescent protein (GFP) ORF under the *p10* promoter. Firstly, a synonym mutation (amino acid 458, aspartic acid; C_1374_ to T_1374_) in the *p74* ORF from AcMNPV (NC_001623.1) was introduced by splicing by overlap-extension PCR using the primers pFwp74Ac, pRevp74Ac, pFwBamAc, and pRevBamAc (Table 1) to add a *BamH*I recognition site between the amino (Nt) and carboxyl (Ct) protein domains. Then, the *p74* coding sequences of AcMNPV (mutated allele), SeMNPV, and HearSNPV-G4 (NC_002169 and NC_002654; both sequences naturally possessing the *BamH*I site in the desired position) were amplified by PCR using the corresponding primer pairs pFwp74Ac/pRevp74Ac, pFwP74Se/pRevP74Se, or pFwP74Ha/pRevP74Ha (Table 1). The PCR products thus obtained were cloned in phase with the 6×His-tag of the pQe30 vector (Qiagen, Hilden, Germany) using *Bg*lII/*BamH*I (for insert or vector, respectively) and *Hind*III endonucleases, T4 DNA ligase (New England Biolabs, Ipswich, MA, USA), and standard procedures to add a common epitope for the immunological detection of recombinant P74s [64]. These recombinant plasmids were used as a source of complete, Nt, or Ct P74 sequence domains to generate different ORF versions under the *polyhedrin* promoter in the pFastBac-Dual-GFP vector (AcAc (Nt and Ct of *p74* from AcMNPV); SeSe (Nt and Ct of *p74* from SeMNPV); HaHa (Nt and Ct of *p74* from HearSNPV); AcSe (Nt of *p74* from AcMNPV and Ct from SeMNPV); AcHa (Nt of *p74* from AcMNPV and Ct from HearSNPV); SeAc (Nt of *p74* from SeMNPV and Ct from AcMNPV); Ac (only Nt of *p74* from AcMNPV); null (without *p74* ORF)) by the use of *EcoR*I, *BamH*I, *Hind*III, T4 DNA ligase, and standard procedures [64]. These constructs (previously certified by Sanger sequencing; STAB VIDA, Caparica, Portugal; Macrogen, Republic of Korea) were used to generate recombinant baculovirus stocks (BacΔp74/GFP + p74AcAc; BacΔp74/GFP + p74SeSe; BacΔp74/GFP + p74HaHa; BacΔp74/GFP + p74AcSe; BacΔp74/GFP + p74AcHa; BacΔp74/GFP + p74SeAc; BacΔp74/GFP + p74Ac; BacΔp74/GFP) employing the DH10bacΔp74 strain and Bac-to-Bac protocols. Furthermore, a non *p74*-knockout version was produced (Bac/GFP) using the original bMON14272 bacmid. All virus stocks were stored at 4 °C and titrated by an end-point dilution assay [65,66].

### 2.5. OB Generation for the Recombinant Baculoviruses

Both bMON14272 and bMON14272Δp74 do not express the polyhedrin gene; therefore, they do not generate OBs in cell culture. Due to this, a co-occlusion strategy was employed to produce infecting OBs using as helper a *p74*-deficient virus that expresses polyhedrin. Thus, a recombinant baculovirus using bMON14272Δp74 and a pFastBac-Dual carrying mCherry ORF (under *p10* promoter) and AcMNPV polyhedrin ORF (under *polyhedrin* promoter) was generated (BacΔp74/Cherry+polH), multiplied, and titrated as previously mentioned to produce BVs and OBs. All OB productions were carried out by coinfecting Sf21 cells with each GFP-producing virus (providing variants of P74) with the mCherry-producing virus (providing polyhedrin) at a multiplicity of infection (MOI) = 5 for each one. After 5 days of incubation at 25 °C, the cells were harvested, pelleted by centrifugation at 1000× *g* for 5 min, and resuspended in 0.1% *w/v* SDS. OBs were separated from the cellular debris by loading the lysate in a 40% *w/v* sucrose solution and centrifuging at 30,000× *g* for 30 min. The resulting pellets were washed with distilled water and centrifuged at 2000× *g* for 5 min. Finally, after resuspending the OB pellets in distilled water, they were quantified using a Neubauer chamber and an optical microscope, thus generating AcMNPV-Δp74, AcMNPV-p74AcAc, AcMNPV-p74SeSe, AcMNPV-p74HaHa, AcMNPV-p74AcSe, AcMNPV-p74AcHa, AcMNPV-p74SeAc, AcMNPV-p74Ac, and AcMNPV-p74wt. The presence of P74 protein in the OBs was verified by Western blot, first resolving the viral proteomes in a 10% SDS polyacrylamide gel electrophoresis (SDS-PAGE) and then using mouse anti-6×HIS as the primary antibody (BD Biosciences, Franklin Lakes, NJ, USA) and a goat anti-mouse immunoglobulin conjugated with HRP as the secondary antibody (Jackson ImmunoResearch Laboratories INC, West Grove, PA, USA). Protein bands were detected using an enhanced chemiluminescent substrate (ECL; Thermo Fisher Scientific, Watham, MA, USA) and a C-Digit blot scanner (LI-COR, Bad Homburg, Germany).

### 2.6. Bioassays with Recombinant AcMNPVs

Two methodologies were used to assess the infectivity of the recombinant viruses. On the one hand, 16 fourth-instar *S. exigua* larvae were injected into the hemocoel with 5 µL of 5 × 10^4^ BVs of each different AcMNPV in MQ water (Grace’s medium was used as a negative control). The infections were confirmed by the visualization of fluorescence (Leica MZ10 F), and larvae were incubated at 26 °C until death. On the other hand, to evaluate the oral infectivity of co-occluded recombinant viruses by the droplet feeding method [67], suspensions of OBs were diluted in a 10% *v/v* phenol red and 10% *w/v* sucrose solution to perform bioassays in 16 early third-instar *S. exigua* or *R. nu* larvae (1 × 10^7^ OBs/mL). Exposed insects were maintained at 26 °C and mortality was recorded every 12 h (*S. exigua*) or 24 h (*R. nu*) until all individuals died. The bioassays were performed side-by-side for all the viruses and repeated three times. Mortality curves were assessed using the Kaplan–Meier method and compared using the log-rank analysis (Mantel–Cox test) using GraphPad Prism software (GraphPad Software Inc., San Diego, CA, USA). The hemolymph was recovered from infected larvae to expose Sf21 cells growing in monolayers on six-well plaques and was incubated for 4 days. The supernatants of infection recovered after this time were treated with PrepMan^®^ Ultra reagent (Applied Biosystems, Waltham, MA, USA) to obtain the viral DNAs that were later used as PCR templates using the primers FwMe53 y RvP74seq (Table 1). These PCR products were sequenced by Sanger method (STAB VIDA, Caparica, Portugal) to confirm the genetic stability of the *p74* knockout.

## 3. Results

### 3.1. P74 Phylogeny and Protein Domains

#### 3.1.1. Phylogeny

**Figure 1:**
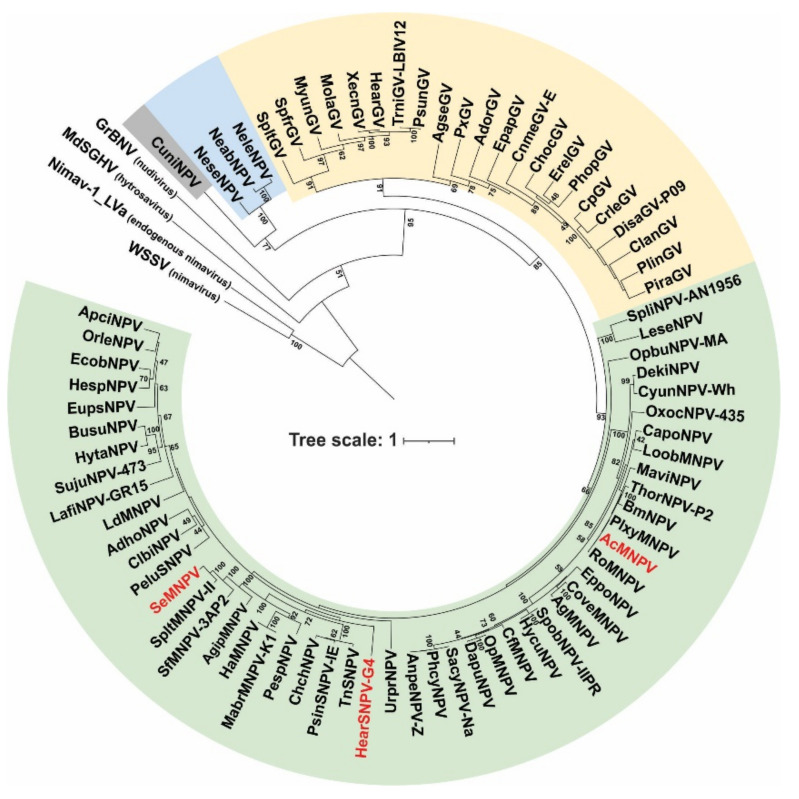
Phylogenetic analysis of the P74 proteins. Evolutionary inference of the P74 protein considering species of the four baculovirus genera and representative species of other invertebrate viruses (nudivirus, hytrosavirus, and nimavirus). Bootstrapping values greater than 40 are shown. Alphabaculoviruses, betabaculoviruses, gammabaculoviruses, and deltabaculoviruses are shaded in green, yellow, blue, and gray, respectively. The three species of alphabaculoviruses considered in the experimental studies are in red letters.

The baculovirus PIF proteins are structural polypeptides associated with the ODV envelope and functionally essential for primary infection. P74 phylogeny reproduced the organization of *Baculoviridae* in the four recognized genera [6], showing an ancestral evolution pattern as the viral family diversified (Figure 1). Orthologs to this protein are found in other ancestrally related invertebrate virus families such as *Nudiviridae*, *Hytrosaviridae*, and *Nimaviridae* [36], suggesting the existence of similar functional roles for this protein in those viruses.

The P74 proteins considered in this work to evaluate their role in the AcMNPV PIF complex belong to well-differentiated clades within alphabaculoviruses. Furthermore, both AcMNPV and SeMNPV can infect *Spodoptera exigua* larvae (one of the models to be evaluated with the P74 variants), whereas HearSNPV cannot.

#### 3.1.2. Protein Domains

**Figure 2:**
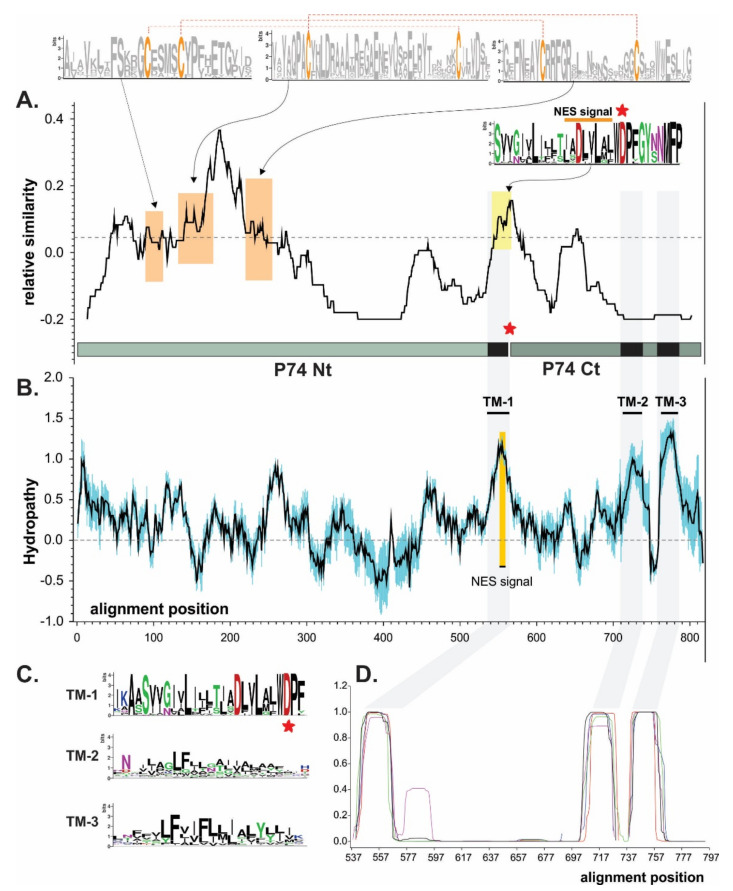
Domain architecture of P74 proteins. Bioinformatics studies for P74 protein consensus based on sequences from species of four baculovirus genera. (**A**) P74 similarity plot. The dashed line indicates the average value. Sequence logos highlight the six cysteines perfectly conserved in all P74 (orange boxes). Dashed red lines show the connectivity predicted with Disulfind server. The putative NES is also indicated (yellow box), and the point of separation between the Nt and Ct regions is marked with a red asterisk. The WDP triad contains a conserved *BamH*I site at the nucleotide sequence level (or the possibility of introducing it through a same-sense point mutation) further used on the construction of hybrid proteins. (**B**) P74 hydropathy profile (black line). Variability was considered as the standard deviation (in blue). The three putative transmembrane regions (TMs) are indicated, with one of them (TM-1) containing a putative NES sequence. (**C**) Sequence logos of each of the TM detected. (**D**) TM predictions. These regions are highlighted in gray in all analyses.

A P74 similarity study considering baculoviruses from the four genera revealed the occurrence of few conserved motifs, where it was possible to highlight three at the amino terminal region (Nt). Each of these three domains contains two cysteines (probably necessary for folding by putative disulfide bonds), along with one at the carboxyl-terminal region (Ct) containing a putative NES which could also contribute to the protein localization on the virion surface (Figure 2A). In this regard, three transmembrane (TM) regions can also be predicted (NES is contained in TM-1) at the Ct, which would allow the polypeptide to be structured in the ODV envelope exposing the Nt outside of the virion (Figure 2B–D) as previously reported [38].

This organization could suggest that the P74 protein has a sector with a structural role in the Ct (containing TMs) and another exposing a functional characteristic in the Nt associated with the specific recognition for the host midgut cells. In fact, it is in the Nt where a protease-mediated activation process has been reported [43,44]. Interestingly, TM-2 and TM-3 differ from TM-1 in that they are in a region of minimal similarity; their physicochemical characteristics seem to be more important than the residues found there. By contrast, TM-1 is in one of the most conserved regions for P74 in *Baculoviridae*. Immediately downstream of the NES (and at the end of the TM-1) appears a conserved amino-acid triad consisting of tryptophan, aspartic acid, and proline (WDP). In this sense, the corresponding nucleotide sequence is “TGG GAT CCN”, enabling the occurrence of a *BamH*I recognition site (--G GAT CC-). This is an opportunity to separate the ORF into two sectors (Nt of 458 vs. Ct of 187 amino acids in AcMNPV) for any baculoviral *p74* (without affecting the amino-acid sequence when in-phase chimerization is performed), thus facilitating a better analysis for this important factor.

### 3.2. AcMNPV Knockout in p74 Gene and Complementation

#### 3.2.1. CRISPR/Cas9 Application

**Figure 3:**
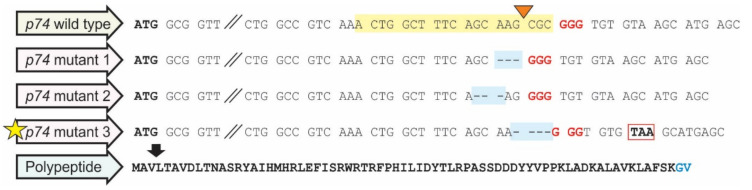
AcMNPV knockout in *p74* gene. Sequence result of the *p74*-knockout AcMNPV-bacmid. The cRNA recognition site is highlighted in yellow, and the protospacer-adjacent motif (PAM) sequence is indicated in red. The dotted line shaded in light blue corresponds to the nucleotide deletion observed. The red box marks the early stop codon generated by the mutation, and the amino acids in blue are substitutions also resulting from the 4 bp deletion. Both initial and stop codons are in bold. The orange triangle shows where the DSB is expected to be generated. Mutant 3 was selected (highlighted with a yellow star).

The AcMNPV bacmid was edited by CRISPR/Cas9 technology, generating a knockout in the *p74* gene by NHEJ. To this end, the Cas9/RNP complex was guided to the initial part of the *p74* ORF (207 bp downstream of ATG codon), and three mutant candidates were selected among 11 viral clones. Two of these affected viruses showed a deletion of 3 bp (nucleotides 205–207 and 203–205), and a third one presented a 4 bp deletion (nucleotides 207–210) in the *p74* ORF. Because the latter evidenced a frameshift that produced an early stop codon (Figure 3), it was the candidate of choice to continue the studies. After verifying the absence of off-target mutations by NGS whole-genome sequencing, this mutagenized bacmid was then used to reconstitute the Bac-to-Bac system to carry out complementation assays with *p74* ORF variants. In this regard, two donor vectors were used to generate two types of viruses over the *p74* knockout backbone: one type expressing P74 variants and GFP; a second type expressing polyhedrin and mCherry. Thus, when cells were simultaneously infected by these recombinant bacmids, orally infective particles could be generated (Figure S1).

#### 3.2.2. AcMNPV OBs with P74 Variants

Regarding the strict host specificity of P74, the corresponding ORFs from SeMNPV and HearSNPV (both species belonging to the group II *Alphabaculovirus* genus; Figure 1) were selected along with the Nt and Ct protein regions considering its separation at the natural *BamH*I site located in WDP, to generate *p74* chimeric alleles for their evaluation in the AcMNPV context (group I *Alphabaculovirus* genus; Figure 1). For the *p74* ORF from AcMNPV, it was first necessary to introduce a same-sense mutation to add a *BamH*I recognition site in the nucleotide sequence that translates into WDP. As shown in the study of relative similarity among P74s of all baculoviral genera (Figure 2A), the protein of AcMNPV has a greater similarity in its Nt domain than in its Ct domain with respect to its orthologs, although both have relatively low identity percentages (Table 2). Another striking fact is the difference in isoelectric point (PI) between the two sectors, with Ct being predominantly acidic (Table S2).

**Figure 4:**
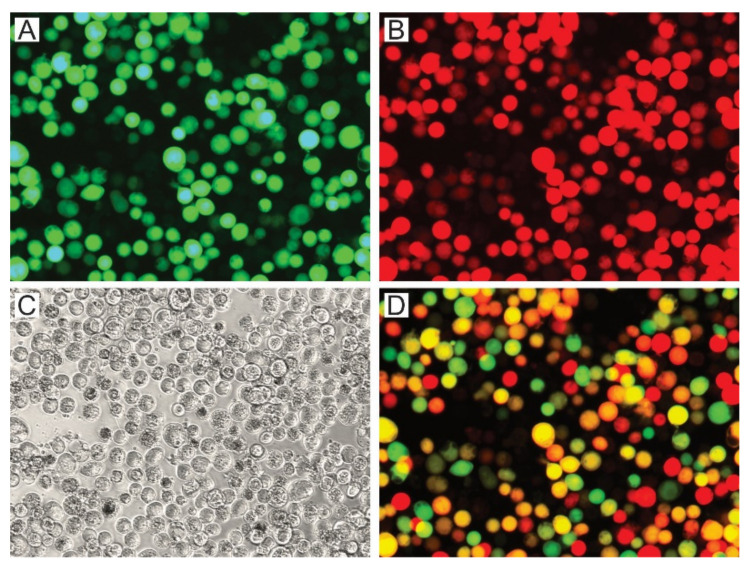
Production of viral OBs with P74 variants. AcMNPV per os infective virions with P74 variants were generated in Sf21 cells by the coinfection of BVs expressing those proteins and GFP, with BVs expressing polyhedrin and mCherry. Both BVs are based on the *p74*-knockout bacmid. Representative microscopy photos (400×) are shown. (**A**) Green fluorescent cells showing that the infection induced GFP expression. (**B**) Red fluorescent cells showing that the infection induced mCherry expression. (**C**) Bright field (the presence of OBs can be seen). (**D**) Merged image obtained through combination of the previous images (**A**,**B**).

Different OB stocks containing variants of P74 (AcMNPV-p74AcAc, AcMNPV-p74SeSe, AcMNPV-p74HaHa, AcMNPV-p74AcSe, AcMNPV-p74AcHa, AcMNPV-p74SeAc, and AcMNPV-p74Ac) and controls (AcMNPV-Δp74 and AcMNPV-p74wt) were generated and produced in Sf21 cells using the knockout bacmid expressing polyhedrin and mCherry to co-occlude the viruses carrying the GFP-P74 versions (Figure S1 and Figure 4).

**Figure 5:**
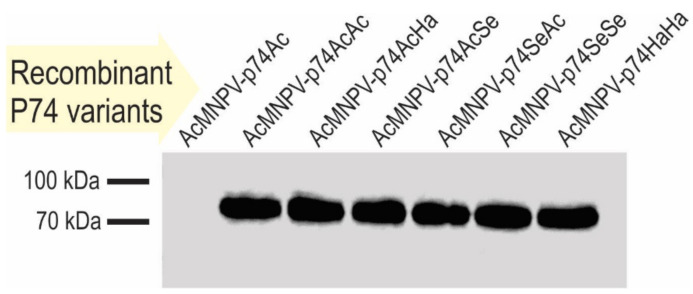
Recombinant P74 protein detection in OBs. Immunodetection of P74 variants in OBs by Western blot using antibodies anti-6×His-tag. The source of the OBs evaluated is indicated on each lane.

The green and red fluorescent cells, perceived as yellow and orange cells in the merged image (Figure 4D), show that the coinfection occurred, and that complemented OBs could be generated. The presence of the P74 recombinant proteins in the OBs was then confirmed by Western blot using the 6×His-tag added at the Nt (Figure 5).

As indicated by the results of cell phenotype (Figure 4), all recombinant OBs could be adequately produced containing the different versions of the P74 protein. Regarding the immunoassay, it was only not possible to detect the 6×His-P74 Nt domain when not fused to any P74 Ct (Figure 5), suggesting that it was probably found in a lower proportion, or that the tag was removed by some proteolytic activity promoted, perhaps, by the absence of the terminal end. This unexpected result was not due to changes in the genetic sequence (this was verified by sequencing); thus, this batch of OBs was also considered in the subsequent assays.

### 3.3. Infectivity of AcMNPV Variants

#### 3.3.1. Infectivity of AcMNPV Variants in *S. exigua*

**Figure 6:**
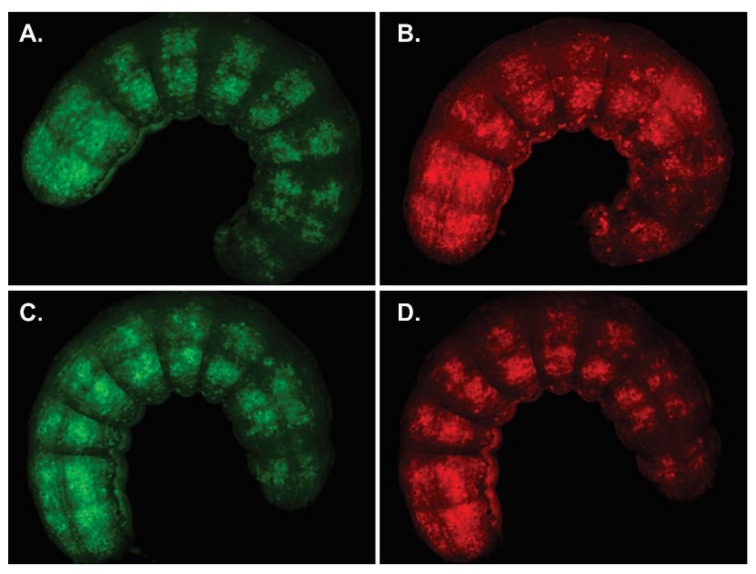
Infectivity of complemented AcMNPV *p74* knockout. *S. exigua* larvae exposed to virions derived from AcMNPV knocked out in *p74* and supplemented with the ORF variant of the same virus, AcMNPV-p74AcAc. (**A**) Representative larva after BV injection showing GFP and (**B**) mCherry expression. (**C**) Representative larva after per os infection with OBs showing GFP and (**D**) mCherry expression. All images (0.8×) were taken 120 h after starting treatment.

The infectivity of all recombinant AcMNPVs was primarily validated by BV injections in *S. exigua* larvae (insect susceptible to oral and intrahemocelic AcMNPV infection) confirming that, for all cases, the secondary infection was not affected (Figure 6A,B). In addition, to control the correct performance of the model under study, OBs of AcMNPV-p74AcAc were employed to per os infect the same invertebrate species (Figure 6C,D).

These studies showed that cell-to-cell infection was not affected, and that the 6×His-tag (and the introduction of the single mutation to add the *BamH*I site in *p74* ORF from AcMNPV) did not prevent the success of the primary infection.

**Figure 7:**
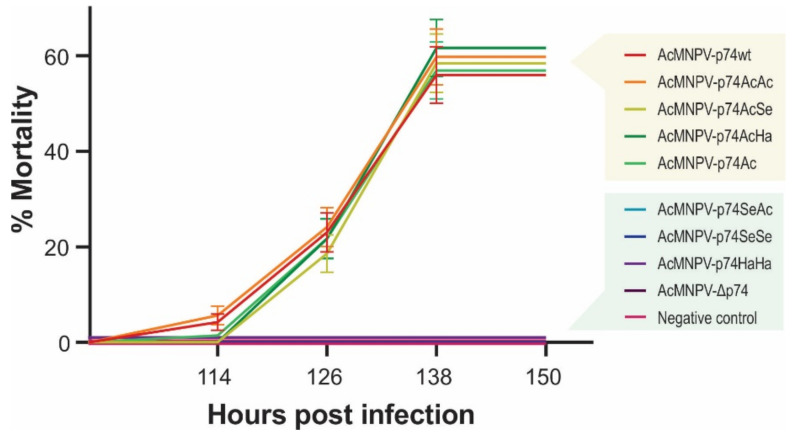
Virulence of the recombinant AcMNPVs in *S. exigua*. Mortality percentages of third-instar larvae of *S. exigua* orally infected by the droplet feeding method with the different variants of OBs (1 × 10^7^ OBs/mL). Bioassay results (48 larvae in experimental units of 16 individuals for each treatment) are shown, and standard deviations are included. Exposed insects were maintained at 26 °C, and mortality was recorded every 12 h.

Comparative bioassays were then carried out to study the influence of the different P74 domains and variants on the viral specificity and pathogenicity (Figure 7).

*S. exigua* larvae showed not to be susceptible to the per os infection with AcMNPV-Δp74, as expected since it does not express any P74, reconfirming its PIF nature as previously reported [18,45]. Thus, the remaining polypeptide of 71 amino acids resulting from NHEJ mutation in *p74* ORF (Figure 3) did not generate an infective phenotype, confirming the gene pseudogenization. In addition, complementation with the P74 protein from SeMNPV (AcMNPV-p74SeSe) and HearSNPV (AcMNPV-p74HaHa) did not produce infectious OBs, regardless of whether this replacement was made from viruses to which *S. exigua* is naturally susceptible (SeMNPV) or not (HearSNPV). In the same direction, AcMNPV-p74SeAc also failed to infect the insects, showing that the Ct domain would not contain the host-restricted functions. However, AcMNPV-p74AcAc, AcMNPV-p74AcSe, AcMNPV-p74AcHa, and AcMNPV-p74Ac were infectious (as well as the positive control, AcMNPV-p74wt). Interestingly, not only were AcMNPV-p74wt and AcMNPV-p74AcAc equally infectious and lethal despite expressing P74 under different promoters (*p74* vs. *polyhedrin* gene promoters, respectively), but also all of those that contained the P74-Nt domain from AcMNPV performed similarly (whether the motif was alone or combined with the Ct region of the other tested species), with no significant differences observed in mortality among them. Although it was not possible to demonstrate the presence of the Nt domain from the AcMNPV-p74Ac OBs by recognizing the fused 6×His-tag by Western blot, this assay showed that it was present to rescue the virus infectivity. In all cases, the viral progeny produced in the larvae where the infection progressed were analyzed to certify the stability of the mutation in *p74* gene.

#### 3.3.2. Infectivity of AcMNPV Variants in *R. nu*

**Figure 8:**
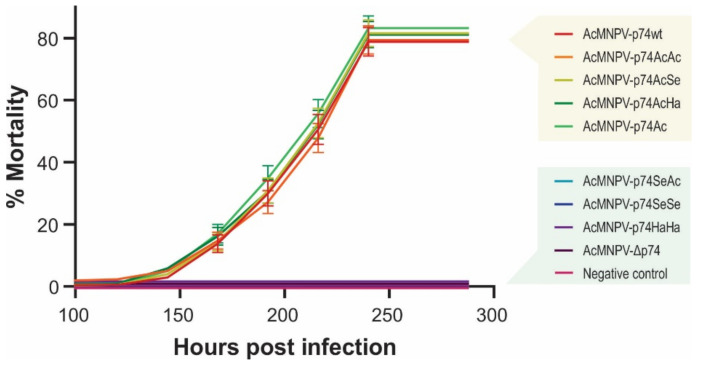
Virulence of the recombinant AcMNPVs in *R. nu*. Mortality percentages of third-instar larvae of *R. nu* orally infected by the droplet feeding method with the different variants of OBs (1 × 10^7^ OBs/mL). Bioassay results (48 larvae in experimental units of 16 individuals for each treatment) are shown, and standard deviations are included. Exposed insects were maintained at 26 °C, and mortality was recorded every 24 h.

To analyze the influence of the host, similar assays were carried out in *R. nu* larvae (neither susceptible nor permissive for SeMNPV and HearSNPV, but orally and intrahemocelic infective for AcMNPV) obtaining equivalent results (Figure 8 and Figure S2).

As observed in *S. exigua* larvae, AcMNPV-p74wt, AcMNPV-p74AcAc, Ac-MNPV-p74AcSe, AcMNPV-p74AcHa, and AcMNPV-p74Ac were infective for *R. nu*, and no significant differences were observed. Furthermore, signs of infection or mortality were not detected for viruses that did not contain the AcMNPV P74 Nt domain, i.e., AcMNPV-p74SeAc, Ac-MNPV-p74SeSe, AcMNPV-p74HaHa, and AcMNPV-Δp74.

## 4. Discussion

Previous studies failed to achieve infective baculoviruses when PIFs were replaced by orthologous sequences [36,68], except for some PIF3 replacements in *Helicoverpa armigera* nucleopolyhedrovirus (HearNPV) by the homologs from *Spodoptera litura* nucleopolyhedrovirus (SpltNPV) and *Mamestra brassicae* multiple nucleopolyhedrovirus (MbMNPV), which retained only partial oral infectivity [47,69], providing evidence of the stringency on the recognition process of the host in the primary infection. This may be due to the stability of the PIF complex in structural terms and/or due to the need for specific interactions with host factors, including cell receptors and activating enzymes such as proteases. This is the first study that analyze the expression of P74 chimeras (combining Nt and Ct domains from different species) during an infection cycle to try to find which region (if not the entire protein) is the one that carries such biological restrictions.

The non-expression of any PIF protein separately nullifies the infectivity of the ODVs in susceptible larvae. This would indicate that there are important structural and functional constraints where each intervening factor is required in an essential way. Three putative transmembrane domains were predicted in P74 protein consistent with previous reports [17,42], although other studies only identified the two closest (TM-2 and TM-3) to the carboxyl terminal end [36,38]. It is not clear whether TM-1 is a real transmembrane region, because truncated versions of P74 (only deleting TM-2 and TM-3 and replacing it with GFP) were soluble and apparently not anchored to the ODV membrane [45]. After being synthesized in the cytoplasm, PIFs must be imported into the nucleus for proper ODV assembly. Although most of the ODV envelope proteins contain an INM-SM sequence which tags them for nuclear import [38,40,70], no similar motif was reported in P74. Nonetheless, in this study, the presence of a NES-like sequence was predicted at the end of TM-1, one of the most conserved regions for P74 in *Baculoviridae*. These kinds of motifs are rich in hydrophobic residues (e.g., leucine), start with an alpha helix, and are usually involved in the transportation of proteins from the nucleus to the cytoplasm, binding with the export karyopherin known as chromosomal region maintenance 1 (CRM1 or exportin 1) [71]. Given the late appearance of the P74 protein during the infection cycle [18], it could be speculated that this region has the binding role needed for the conformation of the PIF complex in association with the hypertrophied nucleus envelope of the infected cell. This could explain why truncated P74 versions lacking TM-1, TM-2, and TM-3 did not rescue oral infectivity, as demonstrated in previous work [45]. Our results showed that a truncated variant of P74 containing TM-1 was sufficient to rescue the infectivity of a P74 defective AcMNPV. This complementation was successful in the absence of TM-2 and TM-3, as well as when that region was replaced by orthologs from viral species of the same genus but different group, as SeMNPV and HearSNPV are. Remarkably, the bioassays also showed no significant differences in the infectivity or pathogenicity of these AcMNPV-P74 Ct domain replacement or missing variants, when compared to each other or even with AcMNPV-p74wt. This demonstrates that the acidic Ct region of 187 amino acids containing the most important TM regions (TM-2 and TM-3) seems to play a nonessential structural role and can be replaced by orthologous segments from other species (that naturally infect the same host or not) without compromising the P74 activity and the OB virulence.

The maintenance of infectivity does not occur when the Nt is replaced (regardless of whether it comes from a viral species infectious for the host), showing that this domain contains the functional restrictions. It is also interesting to note that the Nt supports peptide fusion without affecting its role, in accordance with previous evidence showing that this region undergoes a proteolysis process for its activation [43,44]. These results together indicate that the region of the first 458 amino acids of P74 protein of AcMNPV is sufficient and essential to consolidate a functional PIF complex in the ODV envelopes and is responsible for the host-related restrictions.

It is important to highlight some methodological aspects associated with functional genomics studies to be considered in this context. The introduction of knockout mutations is an indispensable tool for understanding gene functions. In baculoviruses, this type of research is traditionally performed by replacing viral sequences with marker or reporter genes [46,72]. Although this kind of strategy has made it possible to generate substantial knowledge so far, it carries associated risks, such as the introduction of cryptic promoters or the elimination of sequences with other unknown important roles (e.g., miR genes, origins of replication, expression-modulators of neighboring genes, and psi packaging-like elements) that can impact the viral cycle and the virions that are produced (both morphologically and in their infectious capacities). Many of these functional sequences are usually contained within genes; thus, a knockout by replacement or deletion can simultaneously affect several viral functions [73,74,75]. Therefore, the application of CRISPR/Cas9 technology and NHEJ in baculovirus for these purposes appears as a better option due to its lower probability of unwanted side-effects and should now be the preferred tool for this type of genomic study. In this sense, continuing with the work where the use of gene editing mediated by CRISPR/Cas9 is reported for optimizing the use of baculovirus as a protein expression vector [50], the present study demonstrates its usefulness for baculovirus functional genomics research and proposes a methodological detail for its implementation henceforth.

## 5. Conclusions

The P74 protein is an essential factor to support primary baculovirus infection in a susceptible host, without affecting secondary infection. In this work, it was shown that the elimination of the transmembrane region of the terminal carboxyl end of P74 (an acidic part of low amino-acid conservation but structural preservation) does not compromise its function, and that this region can even be replaced by orthologs of species that infect or do not infect the same host. However, considering our results and those of the literature, it seems that a central hydrophobic region (which can be hypothesized as TM and/or as NES) is essential for the activity and presumably necessary to associate this protein with the rest of the PIF complex and ODV envelope. This “hydrophobic core region” requires further research to elucidate its role because it is strikingly conserved in all baculoviral species. Furthermore, this study opens the door for future genetic editing mediated by CRISPR/Cas9 to improve/change the efficiency of the primary infection by replacing regions of the *p74* gene in wild-type baculovirus, thus increasing the biopesticide power in strategies of biologic control for crop pests.

## Figures and Tables

**Table 1:** List of PCR primers.

Primer	Sequence (5′ to 3′)
FwP74seq	GGTTTTAACAGCCGTCGATTTA
RvP74seq	TAAGATGCATTTGTTGTCGAGC
pFwp74Ac	GAAGATCTATGGCGGTTTTAACAG
pRevp74Ac	CCCAAGCTTAAAATAACAAATCAATTG
pRevBamAc	TAACCGAACGGATCCCATAGCGC
pFwBamAc	GCGCTATGGGATCCGTTCGGTTA
pFwP74Se	ATCAGATCTGCTATGCTCACTTTTGTAGAC
pRvP74Se	ATCAAGCTTATTCCGAATAGAGATTGTCGTAC
pFwP74Ha	ATCAGATCTTTCATGTCGAATATCATTTATATAC
pRvP74Ha	ATCAAGCTTATGTGTATAAATTGTGGTACC
FwMe53	GCTTTGAAATGCACAACG

Restriction enzyme site sequences are indicated (underlined). AGATCT (*Bg*lII); AAGCTT (*Hind*III); GGATCC (*BamH*I).

**Table 2:** P74 identity and similarity.

	P74	AcMNPV	SeMNPV	HearSNPV
	**AcMNPV**	100	55	52
**Complete**	**SeMNPV**	82	100	55
	**HearSNPV**	82	82	100
				
	**AcMNPV**	100	59	57
**Nt**	**SeMNPV**	85	100	59
	**HearSNPV**	83	84	100
	**AcMNPV**	100	46	42
**Ct**	**SeMNPV**	78	100	46
	**HearSNPV**	77	77	100

Values are expressed as percentages using whole numbers (“similarity” is shaded in light gray and “identity” is indicated by an uncolored background). “Complete” refers to the full protein, while “Nt” and “Ct” are respectively the amino and carboxyl terminal domains separated in the amino-acid triad WDP (containing at the nucleotide level a *BamH*I recognition site).

## Data Availability

Not applicable.

## Supplementary Materials

The following are available online at https://www.mdpi.com/article/10.3390/v13122416/s1: Figure S1. AcMNPV variants; Figure S2. Infectivity of complemented AcMNPV *p74* knockout; Table S1. Sequences used in the P74 phylogeny inference; Table S2. P74 molecular weight and isoelectric point.
