# Supplementary material for: The Membrane-Anchoring Region of the AcMNPV P74 Protein Is Expendable or Interchangeable with Homologs from Other Species
Source: Viruses. 2021 Dec 2;13(12):2416. doi: 10.3390/v13122416 (PMC8704774; doi:10.3390/v13122416)
Supplement: Supplementary file 1 [file viruses-13-02416-s001.zip › viruses-1473153-supplementary.pdf]

Supplementary Material

Table S1. Sequences used in the P74 phylogeny inference

Species	Isolate	Denomination	GenBank
***Alphabaculovirus* (group I)**			
Antheraea pernyi NPV	Z	AnpeNPV-Z	NC_008035
Anticarsia gemmatalis MNPV	2D	AgMNPV	NC_008520
Autographa californica MNPV	Clone C6	AcMNPV	NC_001623
Bombyx mori NPV	T3	BmNPV	NC_001962
Catopsilia pomona	416	CapoNPV	NC_030240
Choristoneura fumiferana MNPV		CfMNPV	NC_004778
Condylorrhiza vestigialis MNPV		CoveMNPV	NC_026430
Cyclophragma undans NPV	Whiov	CyunNPV-Wh	KT957089
Dasychira pudibunda NPV	ML1	DapuNPV	KP747440
Dendrolimus kikuchii NPV	strain YN	DekiNPV	JX193905
Epiphyas postvittana NPV		EppoNPV	NC_003083
Hyphantria cunea NPV		HycuNPV	NC_007767
Lonomia obliqua MNPV	SP/2000	LoobMNPV	KP763670
Maruca vitrata NPV		MaviNPV	NC_008725
Orgyia pseudotsugata MNPV		OpMNPV	NC_001875
Oxyplax ochracea NPV	435	OxocNPV-435	MF143631
Philosamia cynthia ricini NPV		PhcyNPV	JX404026
Plutella xylostella MNPV	CL3	PlxyMNPV	NC_008349
Rachiplusia ou MNPV		RoMNPV	NC_004323
Samia cynthia NPV	Nagano	SacyNPV-Na	LC375538
Spilosoma obliqua NPV	IIPR	SpobNPV-IIPR	KY550224
Thysanoplusia orichalcea NPV	P2	ThorNPV-P2	NC_019945
***Alphabaculovirus* (group I)**			
Adoxophyes honmai NPV		AdhoNPV	NC_004690
Agrotis ipsilon MNPV		AgipMNPV	NC_011345
Apocheima cinerarium NPV		ApciNPV	NC_018504
Buzura suppressaria NPV	Hubei	BusuNPV	NC_023442
Chrysodeixis chalcites NPV		ChchNPV	NC_007151
Clanis bilineata NPV	DZ1	ClbiNPV	NC_008293
Ectropis obliqua NPV	Strain A1	EcobNPV	NC_008586
Euproctis pseudoconspersa NPV		EupsNPV	NC_012639
Helicoverpa armigera MNPV		HaMNPV	NC_011615
Helicoverpa armigera NPV	Strain G4	HearSNPV-G4	NC_002654
Hemileuca sp NPV		HespNPV	NC_021923
Hyposidra talaca NPV		HytaNPV	MH261376
Lambdina fiscellaria NPV	GR15	LafiNPV-GR15	NC_026922
Leucania separata NPV	Strain AH1	LeseNPV	NC_008348
Lymantria dispar MNPV		LdMNPV	NC_001973
Mamestra brassicae MNPV	K1	MabrMNPV-K1	NC_023681
Operophtera brumata NPV	MA	OpbuNPV-MA	NC_040621
Orgyia leucostigma NPV	CFS77	OrleNPV	NC_010276
Peridroma sp NPV	GR-167	PespNPV	NC_024625

Perigonia lusca SNPV		PeluSNPV	NC_027923
Pseudoplusia includens SNPV	IE	PsinSNPV-IE	NC_026268
Spodoptera exigua MNPV		SeMNPV	NC_002169
Spodoptera frugiperda MNPV	3AP2	SfMNPV-3AP2	NC_009011
Spodoptera littoralis NPV	AN1956	SpliNPV-AN1956	JX454574
Spodoptera litura II MNPV		SpltMNPV-II	NC_011616
Sucra jujuba NPV	473	SujuNPV-473	KJ676450
Trichoplusia ni SNPV		TnSNPV	NC_007383
Urbanus proteus NPV	Southern Brazil	UrprNPV	NC_029997
Betabaculovirus			
Adoxophyes orana GV		AdorGV	NC_005038
Agrotis segetum GV		AgseGV	NC_005839
Choristoneura occidentalis GV		ChocGV	NC_008168
Clostera anachoreta GV	HBHN	ClanGV	NC_015398
Cnapahlocrocis medinalis GV	Strain Enping	CnmeGV-E	NC_029304
Cryptophlebia leucotreta GV		CrleGV	NC_005068
Cydia pomonella GV		CpGV	NC_002816
Diatraea saccharalis GV	Parana-2009	DisaGV-P09	NC_028491
Epinotia aporema GV		EpapGV	NC_018875
Erinnyis ello ello GV	Strain BrS86	ErelGV	NC_025257
Helicoverpa armigera GV		HearGV	NC_010240
Mocis latipes GV	Southern Brazil	MolaGV	NC_029996
Mythimna unipuncta GV	MyunGV#8	MyunGV	NC_033780
Phthorimaea operculella GV		PhopGV	NC_004062
Pseudaletia unipuncta GV	Strain Hawaiin	PsunGV	NC_013772
Pieris rapae GV	Wuhan	PiraGV	NC_013797
Plodia interpunctella GV	Cambridge	PlinGV	NC_032255
Plutella xylostella GV		PxGV	NC_002593
Spodoptera frugiperda GV	VG008	SpfrGV	NC_026511
Spodoptera litura GV	Strain K1	SpltGV	NC_009503
Trichplusia ni GV	LBIV-12	TrniGV-LBIV12	KU752557
Xestia c nigrum GV		XecnGV	NC_002331
Gammabaculovirus			
Neodiprion lecontei NPV		NeleNPV	NC_005906
Neodiprion sertifer NPV		NeseNPV	NC_005905
Neodiprion abietis NPV		NeabNPV	DQ317692
Deltabaculovirus			
Culex nigripalpus NPV	Florida1997	CuniNPV	NC_003084
Nudiviridae			
Gryllus bimaculatus nudivirus		GrBNV	NC_009240.1
Hytrosaviridae			
Musca domestica salivary gland hypertrophy virus		MdSGHV	NC_010671.1
Nimaviridae			
Marsupenaeus japonicus endogenous nimavirus DNA		Nimav-1_LVa	BFCD01000001
White spot syndrome virus strain	MEX2008	WSSV	KU216744

Table S2. P74 Molecular weight and isoelectric point

P74	MW (Da)	PI
**AcMNPV (complete)**	73,885.48	4.90
**SeMNPV (complete)**	74,213.85	4.94
**HearSNPV (complete)**	78,406.81	5.13
**AcMNPV (Nt)**	51,673.47	6.52
**SeMNPV (Nt)**	51,606.36	6.48
**HearSNPV (Nt)**	54,706.64	6.06
**AcMNPV (Ct)**	22,230.02	3.95
**SeMNPV (Ct)**	22,625.51	3.99
**HearSNPV (Ct)**	23,718.18	4.45

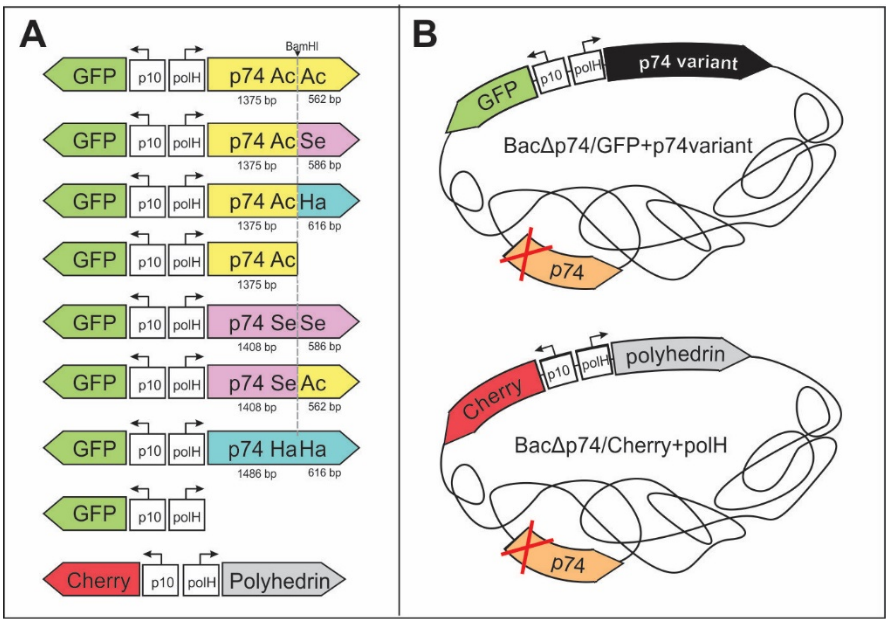

Figure S1. **AcMNPV variants**. The ORF of the *p74* gene of AcMNPV, SeMNPV and HearSNPV were used alone or combining sectors corresponding to the protein amino and carboxyl terminals. **A**. Illustration showing the composition of the different donor plasmids generated containing the *p74* variants and GFP, or polyhedrin and mCherry. Grey dotted line represents the BamHI recognition site between the amino and carboxyl P74 protein domains. **B**. Illustration showing bacmids deficient in the *p74* gene (indel mutation by CRISPR/Cas9 technology and NHEJ) after Bac-to-Bac complementation, one contributing with polyhedrin and the other one with a P74 variant. When both are coinfected in the same cells OBs can be generated with *per os* infective potential.

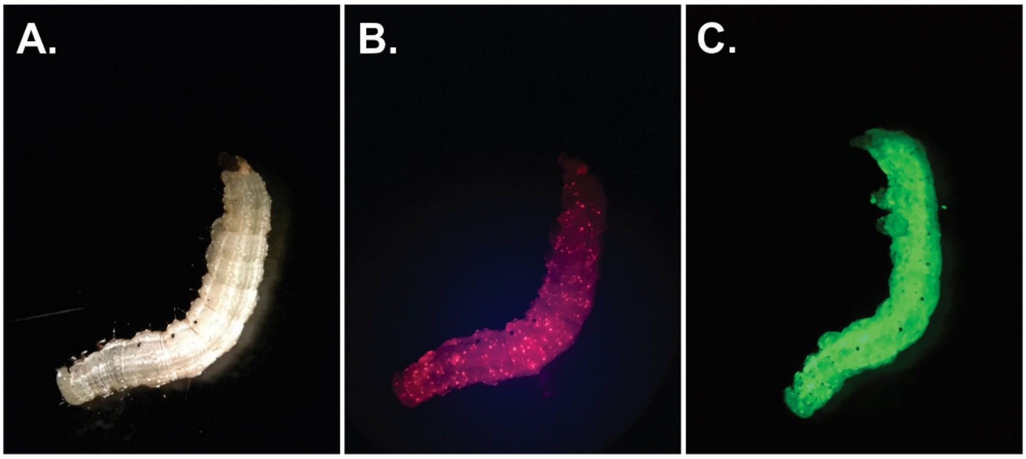

Figure S2. **Infectivity of complemented AcMNPV p74 knockout**. *R. nu* larvae exposed to virions derived from AcMNPV knocked out in *p74* and supplemented with the ORF variant of the same virus carrying the point mutation to introduce the BamHI site. Photographs (0.8X) taken 120 hours after treatment with the virus through *per os* route showing the appearance of a larva in bright field (A.), and the mCherry (B.) and GFP (C.) expression.
